# Does eating behaviour among adolescents and young adults seeking obesity treatment differ depending on sex, body composition, and parental country of birth?

**DOI:** 10.1186/s12889-022-14297-0

**Published:** 2022-10-11

**Authors:** Stephanie E Bonn, Anne Christenson, Helén Eke, Linnea Sjöblom, Anna Dahlgren, Ylva Trolle Lagerros

**Affiliations:** 1 Division of Clinical Epidemiology, Department of Medicine Solna, Karolinska Institutet, Eugeniahemmet T2:02, SE-171 76 Stockholm, Sweden; 2 Center for Obesity, Academic Specialist Center, Stockholm Health Services, Stockholm, Sweden

**Keywords:** Adolescents, Eating behaviour, Obesity, Three factor eating questionnaire, Young adults

## Abstract

**Background:**

Adolescents and young adults around the world experience high rates of weight gain. The underlying eating behaviours that may lead to overconsumption of energy are complex and can depend on a number of factors. The aim of this study was to explore if eating behaviour among adolescents and young adults referred to specialized obesity treatment differed depending on sex, body composition, and parental country of birth.

**Methods:**

Adolescents and young adults aged 16–25 years, referred for obesity treatment in 2018–2021 were included in the study. Eating behaviour was assessed using the Three Factor Eating Questionnaire, comprising domains of uncontrolled-, emotional- and cognitive restraint eating. Student’s t-test was used to compare differences in eating behaviour scores between males and females, and between those having at least one parent born in a Nordic country and those with both parents born outside the Nordic countries. Associations between BMI, waist circumference, and body fat percentage, and eating behaviour as the dependent variable, were examined using linear regression.

**Results:**

A total of 463 participants, mean age 21 years and mean BMI 41.3 kg/m^2^, were included in the analysis. Females scored statistically significantly higher than males on emotional eating (45.8 vs. 35.4, p = 0.002) and cognitive restraint eating (45.4 vs. 39.2, p = 0.009). Participants with at least one parent born in a Nordic country reported a statistically significantly lower score of uncontrolled eating (45.7 vs. 51.3, p = 0.02) compared to participants with both parents born outside the Nordic countries. Further, there were statistically significant inverse associations between cognitive restraint eating scores and BMI (β=-0.64, 95%CI: -0.97 to -0.31), waist circumference (β=-0.44, 95%CI: -0.61 to -0.27) and body fat percentage (β=-0.57, 95%CI: -1.01 to -0.14) in models adjusted for age, sex, smoking, and civil status.

**Conclusion:**

Our findings suggest that sex and parental country of birth may influence eating behaviours among adolescents and young adults referred for specialist obesity treatment. We also found that cognitive restraint eating decreased with increasing BMI, waist circumference, and body fat percentage. This indicates that there may be an inverse association between the ability to restrain oneself from eating and gaining weight, however, the direction of the association must be investigated further. Increased knowledge about eating behaviours may be valuable in the clinical setting.

## Background

Overweight and obesity have increased rapidly during the recent decades and the highest rate of weight gain of any age group is seen among young adults (20–25 years) [1]. According to the World Health Organization, the prevalence of overweight and obesity among children and adolescents age 5–19 has risen from 4% in 1975 to 18% in 2006 [2]. In 2018, the prevalence of overweight and obesity was estimated to be 15% in Swedish children and adolescents aged 11–15 years, while the prevalence was twice as high, 31%, among adolescents and young adults aged 16–29 years [3]. Although obesity-related comorbidities, such as prediabetes and polycystic ovarian syndrome, may be manageable in teenage years, the consequences of obesity usually persist and worsen into adulthood, if there is no weight loss [1, 4].

Obesity occurs as a result of a positive energy balance over time. However, the underlying eating behaviours leading to overconsumption are often complex [5]. Overeating may be a result of a dysregulation of hunger and satiety, but some argue that overeating commonly is a response to negative stimuli or emotions, i.e. emotional eating, and that this is a learned response [6]. Behaviours related to overeating have been associated with higher BMI independent of genotype and shared environment when comparing BMI discordant twin pairs [7]. The heavier twin more often reported higher scores of disinhibition, binge-eating and body dissatisfaction than the leaner twin. Previous studies have also consistently shown positive associations between BMI and body fat percentage, and scores of disinhibited eating, a behaviour that can include elements related to food responsiveness, satiety response and emotional eating [8–11]. The association between BMI and eating behaviours has also been suggested to be population dependent, as it is more pronounced among individuals with obesity than among none-obese subjects [12].

According to previous research, eating behaviours also seem to differ between young men and women [9, 11, 13], but it is less known if this is the case also in treatment seeking adolescents and young adults with obesity. Further, other factors, such as ethnicity and culture, may additionally affect behaviours. For example, it has been shown that dietary behaviours vary greatly across and within different ethnic groups [14], depending on level of education [15] and by socioeconomic status [16–18]. A systematic review established that social and cultural environment, as well as food beliefs and perceptions, predominantly shape dietary behaviours [14]. Social and cultural environment included factors such as cultural identity and a desire to maintain traditional food identity, religious beliefs, and social networks, while food beliefs and perceptions included factors such as beliefs and perceptions of what constitutes healthy foods, parental dietary habits, and the preferences of other family members. Another review by Gilbert et al. [19] found that immigrant generation and age were important factors accounting for changes in dietary habits within some ethic groups following migration to Europe. Alterations in dietary habits, where traditional diets were combined with influences from a more western lifestyle, were more often taking place in younger individuals within the second and third generations of immigrants, as compared to in the first generation that immigrated. Following the increased migration around the world, the number of adolescents and young adults with parents born outside the country of residence is growing in Sweden [20]. The odds of being overweight or obese in childhood are higher among children of North African, Iranian, South American, and Turkish ethnicity compared to children of Swedish ethnicity [21].

To be able to provide effective and individualized clinical treatment, it is important to understand the factors contributing to obesity. Although the time of adolescence and young adulthood is a special period that involves a number of challenges and opportunities, this period is in many ways ideal for weight management and intervention efforts since self-responsibility for health and well-being is under development [22]. Results from a web-based weight loss program showed that an increase in cognitive restraint eating was associated with reductions in weight [23]. Nevertheless, few studies have examined the association between eating behaviour and body composition among adolescents and young adults seeking obesity treatment, or how sex and cultural aspects influence eating behaviour in this group. This kind of knowledge may be key to better success in clinical management of obesity in young people, as a changed eating behaviour may be the primary mechanism to lose weight, independent of type of weight loss treatment.

We aimed to study eating behaviour among adolescents and young adults age 16–25 referred to specialized obesity treatment. Our hypothesis was that eating behaviours would differ depending on sex, parental country of birth, and body composition, although we did not have a pre-defined direction of associations a priori.

## Methods

### Study design and recruitment of participants

This cross-sectional study is part of an ongoing open cohort study, the Swedish Youth with Obesity (SYO) cohort, initiated in 2018 targeting adolescents and young adults aged 16–25 years, being referred for obesity treatment at the Center for Obesity in Stockholm, Sweden. The lowest age limit for referral to the Center for Obesity is 16 years, and the lowest BMI limit is 35 kg/m^2^, or a BMI ≥ 30 kg/m^2^ with at least one co-morbidity. Patients with contraindications, such as a current eating disorder, severe mental health problems, or an ongoing alcohol or drug addiction are not accepted for treatment, and instead referred to other specialists for adequate treatment before obesity treatment can be initiated.

Data presented in this study was collected between March 2018 and December 2021. In conjunction with the invitation letter for the initial appointment at the clinic, everyone was also sent a questionnaire, information about the study, and informed consent. At the day of enrollment, participants brought their filled in questionnaire to the appointments with the physician, the dietician, the physiotherapist and the nurse, who all had a clinical interest in separate sections of the questionnaire. For example, the physician assessed smoking, while the dietician assessed eating behaviour. Patients without contraindications (such as for example binge eating disorder) and who wanted to start obesity treatment were also given additional oral information about the study. Everyone who signed the informed consent were included in the study. The questionnaire was used in the clinical care of all patients, but if they chose to be part of the study, their questionnaire was kept and used for study purposes. Otherwise it was destroyed after the initial appointment at enrollment to the clinic. Inclusion into the study did not affect the possibility to enroll as a patient.

### Inclusion- and exclusion criteria

Inclusion criteria were enrollment as a patient at the Center for Obesity, aged 16–25 years and informed consent. Among the 489 adolescents and young adults who had agreed to participate in the study, 463 participants were included in analysis. We excluded participants who had not filled out the questionnaire (n = 21), who were lacking data from the medical record (n = 1), who had not reported sex being female or male in the questionnaire (n = 3), or who were completely lacking data from the Three Factor Eating Questionnaire (TFEQ) (n = 1).

### Background variables

Characteristics of study participants were collected from the questionnaire. Age at study inclusion was calculated based on birth date and date of responding to the questionnaire. Participants were divided into two groups based on the country of birth of their parents: “Nordic country” or “Other country”. Those reporting that they had at least one parent born in Sweden or other Nordic country (i.e. Denmark, Finland, Iceland, and Norway) were included in the “Nordic country” group, and those reporting that both of their parents were born outside the Nordic countries were included in the “Other country” group. Civil status was categorized as having a partner or not having a partner and smoking was categorized as being a current smoker or not. Based on reported occupation, participants were categorized as students, employed or other, e.g. parental leave, sick leave, unemployed. All participants who had reported studying to some degree were categorized as being students, irrespective of if they also had reported any type of work.

### Clinical variables

Body mass index (BMI, kg/m^2^) was calculated based on height (cm) and weight (to the nearest 0.1 kg) measured by a nurse at the first visit to the Center for Obesity. On the same occasion, waist circumference was measured two fingers above the umbilicus to the nearest cm, and body fat percentage was measured using the body composition monitor Tanita (Model: bc-420MA).

### Three factor eating questionnaire

Eating behaviour was assessed using the validated TFEQ R-21 [12, 24]. This version of the TFEQ comprise 21 questions assessing eating behaviour in three different domains: uncontrolled eating (9 items), emotional eating (6 items), and cognitive restraint eating (6 items). In brief, uncontrolled eating represents the tendency to lose control over ones eating when feeling hungry or being exposed to external stimuli, emotional eating represents the tendency to overeat in response to negative emotions (e.g. feeling depressed, anxious or lonely), and cognitive restraint eating represents the individual’s conscious control over food intake [10, 25].

At least 50% of the questions in a domain had to be answered in order to obtain a score for that domain. Item values were transformed to raw scale scores for each domain by calculating the mean of all items included in the scale multiplied with the number of items in the scale. Thereafter, a score ranging between 0 and 100 was calculated for each domain, representing a percentage of the total possible scale score within the domain. A higher score indicates more of that certain behaviour [12].

### Statistical analysis

Characteristics of our study population are presented as mean and standard deviations (SD) or n and percent, for continuous and categorical variables, respectively. Variables were checked for completeness and outliers. Little’s chi-square test was performed using the mcartest command in STATA to test the assumption of missing completely at random (p = 0.12). Continuous variables were additionally checked for normality. Student’s t-test were used to compare differences in TFEQ scores between males and females, and between participants with at least one parent born in a Nordic country and participants with both parents born outside the Nordic countries. Associations between sex, parental country of birth, BMI, waist circumference, and body fat percentage, and eating behaviour as the dependent variable were examined using linear regression. Models for the association between sex and eating behaviour were adjusted for age (model 1), and for age, smoking, civil status and parental country of birth (model 2), while models for the association between parental country of birth and eating behaviour were adjusted for age and sex (model 1), and for age, sex, smoking, and civil status (model 2). In models for BMI, waist circumference and body fat percentage, adjustments were made for age and sex (model 1) and for age, sex, smoking, civil status, and parental country of birth (model 2). Included confounders were selected based on prior subject matter knowledge of the potential underlying causal relationships (direction of associations) between selected confounding factors and the exposure and outcome variables in question. All of the selected covariates were statistically significantly associated to at least one of the three eating behaviour constructs when analyzed independently using linear regression. A p-value < 0.05 was considered statistically significant. All analyses were run in STATA 14.2 (Stata Corporation, College Station, TX, USA).

## Results

Characteristics of our study population are presented in Table 1. Participants were on average 21 years old and had a mean BMI of 41.3 kg/m^2^. More females (76.7%) than males were included in the study and the majority of participants (57.0%) reported having at least one parent born in a Nordic country. Most of the participants were single (67.0%), students (58.1%), and non-smokers (70.6%). The average scores within the three domains of TFEQ were 48.1 (uncontrolled eating), 43.4 (emotional eating), and 43.9 (cognitive restraint eating).

**Table 1:** Characteristics of the study group (n = 463)

			n	Mean	(SD)
**Age**, years			463	21.0	(3.0)
**BMI**, kg/m^2^			463	41.3	(6.0)
**Waist circumference**, cm			370	116.0	(13.9)
**Body fat percentage**			276	46.2	(6.6)
**Blood pressure**					
	Systolic		447	119.9	(12.1)
	Diastolic		447	75.9	(9.7)
**TFEQ**					
	Uncontrolled eating		463	48.1	(25.5)
	Emotional eating		463	43.4	(30.2)
	Cognitive restraint eating		463	43.9	(21.5)
				**n**	**(%)**
**Country of birth parents**			463		
		Nordic country^1^		264	(57.0)
		Other country^2^		199	(43.0)
**Civil status**			463		
		No partner		310	(67.0)
		Partner/married		136	(29.4)
		Other/missing data^3^		17	(3.7)
**Occupation**			463		
		Student		269	(58.1)
		Employed		137	(29.6)
		Other^4^		57	(12.3)
**Smoker** ^**3**^			456		
		No		322	(70.6)
		Yes		134	(29.4)

^1^At least one parent born in a Nordic country; ^2^Both parents born outside the Nordic countries; ^3^ n = 7 missing; ^4^E.g. parental leave, sick leave, unemployed

Table 2 shows results from the TFEQ stratified by sex and parental country of birth. Female participants scored significantly higher than males on the TFEQ within the domains of emotional eating (45.8 vs. 35.4, p = 0.002) and cognitive restraint eating (45.4 vs. 39.2, p = 0.009). There was no difference in mean score of uncontrolled eating between males and females. When comparing participants with at least one parent born in a Nordic country to participants with both parents born outside the Nordic countries, those belonging to the Nordic group reported a significantly lower score of uncontrolled eating (45.7 vs. 51.3, p = 0.02). No differences in emotional or cognitive restraint eating were seen depending on parental country of birth. Results from adjusted linear regression models (Table 3) are in agreement with results from the t-tests. Females had statistically significantly higher scores of emotional eating (β = 10.72, 95% CI: 4.03 to 17.40) and cognitive restraint eating compared to males (CR β = 6.06, 95% CI: 1.32 to 10.80). Those with both parents born outside the Nordic countries had a statistically significantly higher score of uncontrolled eating (β = 5.64, 95% CI: 0.92 to 10.36) compared to adolescents and young adults with at least one parent born in a Nordic country.

**Table 2:** Difference in TFEQ-scores between sexes and categories of parental country of birth

	Sex				
**TFEQ**	**Male** (n = 108)		**Female** (n = 355)		
	mean	(SD)	mean	(SD)	p-value^1^
Uncontrolled eating	46.6	(24.0)	48.5	(25.9)	0.50
Emotional eating	35.4	(29.3)	45.8	(30.1)	**0.002**
Cognitive restraint eating	39.2	(20.1)	45.4	(21.7)	**0.009**
	**Parental country of birth**				
	**Nordic** ^**2**^ (n = 264)		**Other** ^**3**^ (n = 199)		
	mean	(SD)	mean	(SD)	p-value^1^
Uncontrolled eating	45.7	(24.8)	51.3	(26.0)	**0.02**
Emotional eating	41.9	(30.5)	45.3	(29.9)	0.23
Cognitive restraint eating	43.8	(21.4)	44.1	(21.6)	0.90

^1^Double-sided t-test; ^2^At least one parent born in a Nordic country; ^3^Both parents born outside the Nordic countries

We found statistically significant associations between BMI, waist circumference and percentage of body fat with scores of cognitive restraint eating (Table 3). In fully adjusted models, there was a 0.64-point decrease in cognitive restraint eating score for every one unit increase in BMI (95%CI: -0.97 to -0.31). For every one cm increase in waist circumference, there was a decrease of 0.44 in the score (95%CI: -0.61 to -0.27), and for every 1% increase in body fat, the score decreased by 0.57 (95%CI: -1.00 to -0.14). There was a borderline statistically significant association between waist circumference and emotional eating, with an increase of 0.24 (95%CI: -0.003 to 0.49). We found no other statistically significant associations between BMI, waist circumference, and percentage of body fat with scores for uncontrolled eating or emotional eating.

**Table 3:** Results from linear regression models between BMI, waist circumference, and body fat percentage, and eating behaviour as the dependent variable

		Model 1		Model 2	
		β	**(95% CI)**	**β**	**(95% CI)**
**Sex - Female** ^**1**^					
	Uncontrolled eating	2.71	(-2.80 to 8.23)	2.07	(-3.51 to 7.65)
	Emotional eating	**10.75**	**(4.23 to 17.27)**	**10.72**	**(4.03 to 17.40)**
	Cognitive restraint eating	**5.88**	**(1.24 to 10.52)**	**6.06**	**(1.32 to 10.80)**
**Parental country of birth - Other** ^**2**^					
	Uncontrolled eating	**5.29**	**(0.63 to 9.96)**	**5.64**	**(0.92 to 10.36)**
	Emotional eating	3.22	(-2.32 to 8.76)	3.31	(-2.33 to 8.96)
	Cognitive restraint eating	0.34	(-3.61 to 4.29)	0.89	(-3.11 to 4.90)
**BMI** ^**3**^					
	Uncontrolled eating	-0.05	(-0.45 to 0.35)	-0.02	(-0.42 to 0.37)
	Emotional eating	0.25	(-0.22 to 0.72)	0.28	(-0.20 to 0.75)
	Cognitive restraint eating	**-0.62**	**(-0.95 to -0.29)**	**-0.64**	**(-0.97 to -0.31)**
**Waist circumference** ^**3**^					
	Uncontrolled eating	0.12	(-0.09 to 0.32)	0.14	(-0.07 to 0.35)
	Emotional eating	**0.25**	**(0.006 to 0.49)**	0.24	(-0.003 to 0.49)
	Cognitive restraint eating	**-0.43**	**(-0.60 to -0.26)**	**-0.44**	**(-0.61 to -0.27)**
**Body fat percentage** ^**3**^					
	Uncontrolled eating	-0.22	(-0.74 to 0.30)	-0.18	(-0.70 to 0.34)
	Emotional eating	0.04	(-0.58 to 0.67)	0.07	(-0.56 to 0.70)
	Cognitive restraint eating	**-0.52**	**(-0.95 to -0.09)**	**-0.57**	**(-1.00 to -0.14)**

^1^Model 1 adjusted for age, Model 2 adjusted for age, smoking, civil status and parental country of birth;

^2^Model 1 adjusted for age and sex, Model 2 adjusted for age, sex, smoking, and civil status;

^3^Model 1 adjusted for age and sex, Model 2 adjusted for age, sex, smoking, civil status, and parental country of birth

## Discussion

In this clinical sample of adolescents and young adults referred for specialist obesity treatment in Sweden, eating behaviour was associated with sex, parental country of birth and anthropometric measurers. Participants scored high within all domains of eating behaviour, although females scored higher than males on emotional- and cognitive restraint eating. Having two parents born outside of the Nordic countries also increased the likelihood of scoring high in uncontrolled eating. Furthermore, anthropometrics measured by BMI, waist circumference, and body fat percentage were inversely associated with cognitive restraint eating, indicating that gaining less weight prior to obesity treatment was associated with a higher ability to restrain oneself from eating.

Previous research regarding eating behaviours among adults has shown diverse results. In line with our results, both Gade et al. [26] and Cornelis et al. [27] found that women with obesity reported higher levels of emotional eating compared to men. Contrary to our results where we found no associations between emotional- or uncontrolled eating with BMI, Cornelis et al. [27] found associations between both of these constructs and BMI in both men and women. Nevertheless, results from our fully adjusted models showed a borderline statistically significant association between waist circumference and emotional eating. Additionally, Loffler et al. [28] showed associations between uncontrolled eating and BMI in both men and women. A possible explanation for the discrepancy between our results and those by Cornelis et al. and Loffler et al., could be that their study populations differ from ours. In addition to a much lower mean BMI (25–27 kg/m^2^), the mean ages of their study participants were 67 and 55 years, respectively, compared to ours of 21 years. Karlsson et al. [24] and Czeglédi et al. [29] assessed eating behaviour in populations with obesity. Both studies found that emotional eating was associated with BMI, but Karlsson et al. only found this among women. Their populations were however adults with a mean age of around 50 years, and only Czeglédi et al. used a clinical sample of weight loss patients.

Similar to our findings, Williamson et al. [30] found that a higher score of cognitive restraint eating was associated with lower BMI, but they only included women in their study. In contrast, other studies [25, 27] found that high scores of cognitive restraint eating were related to a high BMI. The latter has also been shown in samples comprising young adults [13, 31]. In a sample of 17 to 20-year-olds, Angle et al. [31] showed that higher levels of both cognitive restraint eating and emotional eating were associated with a higher BMI. Kowalkowska et al. [13], found similar results in a study on university students (mean age 21.3 years) where cognitive restraint eating was positively correlated with BMI in both males and females. However, both of these studies comprised young adults with an average BMI corresponding to normal weight (i.e. BMI < 25 kg/m^2^). Previous studies indicate that the association between eating behaviour and BMI may differ depending on BMI status. Cappelleri et al. [12] showed an association between cognitive restraint eating and BMI in subjects with a BMI ≥ 27 kg/m^2^, while no association was seen among subjects with a BMI < 27 kg/m^2^. Johnson et al. [32] also concluded in a review that the association between cognitive restraint eating and body weight differed between normal weight and obese populations. In normal weight groups, higher scores of cognitive restraint eating was associated with higher body weight, but the opposite was seen in obese groups. This may explain why our results differ from those in previous studies mainly comprising subjects without obesity.

Studies comprising adolescents or young adults are less common than studies using adult populations and it should be kept in mind that the age range for the definition of young adults differs widely between studies. In line with our results, a French study among adolescents and young adults age 14–27 years, also showed that females scored higher within emotional eating and cognitive restraint eating, but showed lower scores of uncontrolled eating, compared to males [33]. Their age range is similar to the one in our study where adolescents and young adults age 16–25 years were included. Another cohort of young adults (aged 20–39 years) [11], identified gender differences regarding both disinhibition, i.e. uncontrolled eating, and restraint scores, where women reported significantly higher scores compared to men. However, it should be noted that the mean BMI indicated that most participants were normal weight. Similar results have been seen in other studies comprising normal weight college students, where females have reported higher scores of cognitive restraint eating and emotional eating compared than males [9, 13]. In the latter study by Kowalkowska et al. [13], authors also found that males had significantly higher uncontrolled eating than females, which was not seen in our sample of adolescents and young adults with obesity.

The influence of other people, including family members, has been shown to be an important correlate of the risk of developing obesity [34]. Compared to native-born populations in Europe, many immigrant-origin groups have been found to have a higher prevalence of diet-related non-communicable diseases, such as obesity, type 2 diabetes and cardiovascular diseases [2, 14]. A study comprising children in Sweden also showed that parental ethnicity affected the odds of being overweight or obese in early childhood [21]. In our study, adolescents and young adults with both parents born outside the Nordic countries scored higher within the domain of uncontrolled eating compared to those who had at least one parent born in a Nordic country. This may reflect a cultural difference in eating habits that might impact the risk of developing obesity and its comorbidities. Knowledge of a possible higher risk of uncontrolled eating in patients with parents from non-Nordic countries may be used to plan and perform treatment and prevention programs.

A strength of this study is the relatively large-sample size comprising adolescents and young adults with obesity. Our population also include individuals who are less likely to participate in research studies, for example individuals with lower socioeconomic status or individuals who have parents born abroad [35]. The sample size enabled stratification on Nordic vs. non-Nordic parental country of birth, allowing us to study if eating behaviours differ depending on different cultural backgrounds. However, we did not have power to stratify on separate countries or continents, since more than 50 countries around the world were represented.

A limitation to our study is that all study participants were recruited among persons interested in losing weight. This may limit the generalizability of our results to all young persons with obesity. Hence, we may have a selected group that already tried to change their eating behaviour to lose weight before being referred to a specialist center. On the other hand, as the analysed data is collected as part of the standard clinical routine, the results may be directly applicable to the daily work with adolescents and young adults seeking obesity treatment. Further, as only patients without contraindications, such as a binge eating disorder, are accepted for treatment and thus, invited to the study, this may have introduced selection bias, although our study sample is likely representative of the patients accepted for treatment. The results give insights and enables improvements for a more efficient and individualized care. Further, even though the majority of the participants were females, which can be considered a limitation, more than a hundred young males were included in the study. This is a strength as studies on young males with obesity are lacking [36]. The distribution of females and males in our study reflects that of the adolescents and young adults enrolled to the clinic. Hence, our population reflects adolescents and young adults 16 years or older seeking obesity care in general. However, in order to understand and fill out the questionnaire, the patient had to be sufficiently fluent in Swedish. This may limit generalizability as those recently immigrated to Sweden, who were still in need of an interpreter, were not sent a questionnaire and informed consent ahead of the initial meeting at the center. Nevertheless, almost half of the cohort had two parents born outside the Nordic countries.

Eating behaviour was self-reported using the TFEQ, which limits our results to the specific constructs assessed by this questionnaire. Nevertheless, the TFEQ has shown acceptable construct validity in adults with obesity [24]. A Finnish study also showed good structural validity of the questionnaire in a large sample (n = 2,943) of young females (17 to 20 years) with a wide range of BMI (14.4 to 49.8 kg/m^2^) [31]. Nevertheless, the majority of participants in our study are 18 years or older, making results from our study more comparable to those of other studies comprising adult populations. Self-reported data may be a limitation if there is a perceived advantage with a specific answer, or if there is a difference in how eating behaviours are reported depending on sex, parental country of birth, or body composition. Social desirability has been associated with self-reported eating behaviours in young adults attending university, especially among young female students [13]. A limitation to our study is that we have not assessed social desirability. However, one could speculate that social desirability may be less prominent in a sample that is actively seeking treatment for obesity, since they have much to gain from reporting their true eating behaviours.

Moreover, due to the Covid-19 pandemic, some of the healthcare visits were digital. Thus, certain measurements of body composition, such as waist circumference and body fat percentage, are missing for a number of participants. The data is, however, to the best of our knowledge, and as indicated by the Little’s chi-square test, randomly missing during this specific time period. Therefore, we believe that, if anything, it would likely cause an attenuation of results due to loss of power.

It should also be noted that since this study is of cross-sectional design, the direction of associations could not be determined. Nevertheless, parental country of birth does per definition precede factors present within the offspring. How eating behaviour in this population of adolescents and young adults is affected by obesity treatment would be interesting and valuable to explore in future studies.

## Conclusion

Sex and cultural heritage seem to influence eating behaviours among adolescents and young adults referred for specialist obesity treatment, and should be considered when planning future weight loss treatment. Adolescents and young adults with different eating behaviours may benefit from being treated by different clinical professions depending on what type of behaviour they display. For example, uncontrolled eating triggered by hunger may be best treated by a dietician, while emotional eating could be targeted by a psychologist. A diversity of cultural background among clinical professionals treating adolescents and young adults seeking obesity care might also increase understanding and improve treatment in adolescents and young adults with different cultural backgrounds.

Scores of cognitive restraint eating also decreased with increasing BMI, waist circumference and body fat percentage. This indicates that gaining less weight may be associated with a higher ability to restrain oneself from eating. However, the direction of such an association must be further investigated. Nonetheless, we believe that our results could be of value for clinicians working with obesity treatment in adolescents and young adults.

## Data Availability

Data from the described cohort are not publicly available due to ethical restrictions. Data used in analysis of this study are available from the corresponding author on request.
